# Women’s Experiences With Family Planning Under COVID-19: A Cross-Sectional, Interactive Voice Response Survey in Malawi, Nepal, Niger, and Uganda

**DOI:** 10.9745/GHSP-D-22-00063

**Published:** 2022-08-30

**Authors:** Aurélie Brunie, Gwyneth Austin, Jamie Arkin, Samantha Archie, Dinah Amongin, Rawlance Ndejjo, Saujanya Acharya, Basant Thapa, Sarah Brittingham, Grace McLain, Philip Mkandawire, Maimouna Hallidou Doudou, Ndola Prata

**Affiliations:** a FHI 360, Washington DC, USA.; b FHI 360, Durham, NC, USA.; c Viamo, Nairobi, Kenya; Now with AInfluence Inc, Nairobi, Kenya.; d Makerere University School of Public Health, Kampala, Uganda.; e Viamo, Kathmandu, Nepal.; f FHI 360, Kathmandu, Nepal.; g PSI, Lilongwe, Malawi.; h Evidence for Sustainable Human Development Systems in Africa, and Université Africaine Privée pour le Développement, Niamey, Niger.; i Evidence for Sustainable Human Development Systems in Africa, University of California, Berkeley, CA, USA.

## Abstract

**Introduction:**

We conducted an assessment in Malawi, Nepal, Niger, and Uganda to document access-related reasons for not using contraceptive methods during the COVID-19 pandemic that led to unintended pregnancies, describe use of modern contraception among women in potential need of contraception compared to before the pandemic, examine method choice, and describe barriers to contraceptive access and use.

**Methods:**

Between December 2020 and May 2021, we conducted an opt-in phone survey with 21,692 women, followed by an outbound survey with 5,124 women who used modern nonpermanent contraceptive methods or who did not want to get pregnant within 2 years but were not using a modern contraceptive method. The surveys examined current behaviors and documented behaviors before the pandemic retrospectively. We used multivariable logistic regression models to examine factors associated with contraceptive use dynamics during COVID-19.

**Results:**

Pregnant women surveyed reported that the pandemic had affected their ability to delay or avoid getting pregnant, ranging from 27% in Nepal to 44% in Uganda. The percentage of respondents to the outbound survey using modern contraception decreased during the pandemic in all countries except Niger. Fear of COVID-19 infection was associated with discontinuing modern contraception in Malawi and with not adopting a modern method among nonusers in Niger. Over 79% of surveyed users were using their preferred method. Among nonusers who tried obtaining a method, reasons for nonuse included unavailability of the preferred method or of providers and lack of money; nonusers who wanted a method but did not try to obtain one cited fear of COVID-19 infection.

**Conclusion:**

We found evidence of surveyed women attributing unintended pregnancies to the pandemic and examples of constraints to contraceptive access and use on the supply and demand side. The effects of the pandemic must be interpreted within the local contraceptive, health system, and epidemiological context.

## Abstract (precis)

Surveyed women attributed unintended pregnancies to COVID-19 and reported constraints to contraceptive access and use in Malawi, Nepal, Niger, and Uganda.

## Teaser

**Key Findings:**

- Between 27% and 44% of pregnant women surveyed in each country indicated that the pandemic affected their ability to delay or avoid pregnancy.
- Fewer surveyed women in potential need of contraception reported current use of modern contraception compared to before the pandemic in all countries except Niger.
- Supply-side constraints, including service closures and product shortages, were among the most common barriers to contraceptive access and use reported by women. Fear of coronavirus disease (COVID-19) infection and lack of money also constrained access and use.
- Results linked the unavailability of one’s preferred method to nonuse and unintended pregnancies, indicating that elasticity of demand is limited.

**Key Implications:**

- Experiences with family planning services during the COVID-19 pandemic provide an opportunity to learn lessons to improve preparedness for future crises.
- Policy makers should incorporate multimonth supply of contraceptives to clients in guidance documents.
- Program managers should consider strategies to ensure continued availability of the full method mix during crises, particularly since disruptions may go unnoticed.
- Program managers should strengthen the provision of services through alternative service delivery channels to mitigate service disruptions.

## INTRODUCTION

Officially declared a pandemic by the World Health Organization (WHO) on March 11, 2020, coronavirus disease (COVID-19) continues to cause disruption around the world. Beyond COVID-19’s direct toll on morbidity and mortality, the pandemic has placed a strain on essential health services, including family planning (FP) services, particularly in low- and middle-income countries (LMICs) where health systems are more fragile. For example, WHO multicountry surveys on continuity of essential health services during the COVID-19 pandemic indicate that 68% of 102 countries reported disruptions to FP services between May and July 2020, and 44% of 104 countries between January and March 2021, with 9% of countries in 2020 and 5% in 2021 reporting a decline above 50% in service use.^1^^,^^2^ In both cases, FP was the most commonly disrupted service in reproductive, maternal, newborn, child, and adolescent health, as well as nutrition. Supply-side disruptions include decreased service availability owing to health facility closure, staff redeployment at the clinic and community level, and contraceptive shortages.^1^^–^^3^ Additionally, clients feared being infected with COVID-19 during care or experienced economic hardship from loss of income, while mobility restrictions such as transport lockdowns hindered access.^1^^,^^2^^,^^4^

While modeling exercises predicted a decline in contraceptive use and a rise in unintended pregnancies,^5^^,^^6^ evidence on the effects of the pandemic on contraceptive behaviors is still emerging. Analyses of population-level data from Performance Monitoring for Action (PMA) surveys in 4 geographies show minimal effects on contraceptive coverage as of May–July 2020.^7^ Individual-level analyses of PMA data in Burkina Faso and Kenya in the same time frame indicate that most contraceptive users sustained use and that more women adopted than discontinued contraception.^4^ Governments and programs have turned to health system data to examine trends in FP services; however, such analyses are challenged by inconsistent reporting, especially during lockdown periods. Moreover, little information is available on women’s perspectives on challenges they experience in making contact with and using services that would assist countries to make informed decisions to ensure continuity of care.

This study aims to expand evidence on women’s experiences with contraceptive access and use in LMICs during the pandemic to identify potential gaps and inform programmatic and policy adjustments. Our focus is not on estimating the effects of the pandemic but on generating evidence from the perspectives of women to illustrate the various ways in which the pandemic may be affecting their journey as they attempt to access and use FP services. This assessment was conducted in Malawi, Nepal, Niger, and Uganda to assist these countries in meeting their commitment to ensure that the FP needs of women and couples continue to be met.

Our article’s focus is not on estimating the effects of the pandemic but on generating evidence from women’s perspectives to illustrate the ways in which the pandemic may be affecting their journey as they attempt to access and use FP services.

Modern contraceptive prevalence among all women of reproductive age (15–49 years) is 45% in Malawi, 33% in Nepal, 29% in Uganda, and 15% in Niger.^8^^–^^11^ The most popular short-acting modern contraceptives are injectables in Malawi (50% of modern users), Nepal (21%), and Uganda (37%), and pills in Niger (40%).^8^^–^^11^ The share of long-acting reversible contraceptives (LARCs) in the method mix is 36% in Uganda, almost 20% in Malawi and Niger, and 11% in Nepal, while permanent contraception represents 5%, 18%, 1%, and 47%, respectively.^8^^–^^11^ Malawi, Niger, and Uganda started implementing response measures to COVID-19 in March 2020, and Nepal began in April 2020. While there were no official lockdowns in any of the study sites during data collection, the environment was characterized by supply chain disruptions, income loss, and access to vaccines in all of the contexts.^12^^–^^14^ A prohibitory order was implemented in Nepal’s Kathmandu valley from April 2021 to June 2021. Specific study objectives were to (1) document access-related reasons for not using contraceptive methods that led to unintended pregnancies, (2) describe the use of modern contraception among women in potential need of contraception compared to before the pandemic started, (3) examine women’s ability to obtain their preferred method, and (4) describe barriers to contraceptive access and use.

## METHODS

### Study Design

We conducted a prospective cohort study with 3 rounds of data collection to document women’s experiences over time. Country selection was guided by partner presence and available resources, with a desire to represent different geographic regions. This article draws on the first round of cross-sectional surveys conducted in Malawi (February 2, 2021–March 18, 2021), Nepal (February 2, 2021–May 4, 2021), Niger (February 2, 2021–May 14, 2021), and Uganda (December 22, 2020–March 31, 2021) to retrospectively examine women’s experiences related to FP services since the beginning of the pandemic. After consultations with country teams, we defined the beginning of the pandemic as March 2020 in Malawi, Niger, and Uganda and April 2020 in Nepal.

We recruited participants through Viamo’s 3-2-1 service, a toll-free, interactive voice response service allowing callers to access voice messages on various topics that are organized into “channels” and presented in local languages.^15^ All callers accessing information through the 3-2-1 service (excluding the topics of news, agriculture, nutrition, malaria, and WASH in Niger due to contractual terms) heard a survey recruitment message followed by a consent statement. Data collection included a short survey (referred to as 3-2-1 survey) with female Viamo 3-2-1 callers aged 18 to 49 years, followed within 1 week by an outbound survey with the subset of participants with a potential need for modern contraception, defined here as including (1) nonpregnant women who reported using nonpermanent modern contraception, and (2) nonpregnant women who reported using a traditional method or not using any form of contraception and that they did not want to get pregnant in the next 2 years (Figure). Participation was restricted to once per phone number.

**FIGURE:**
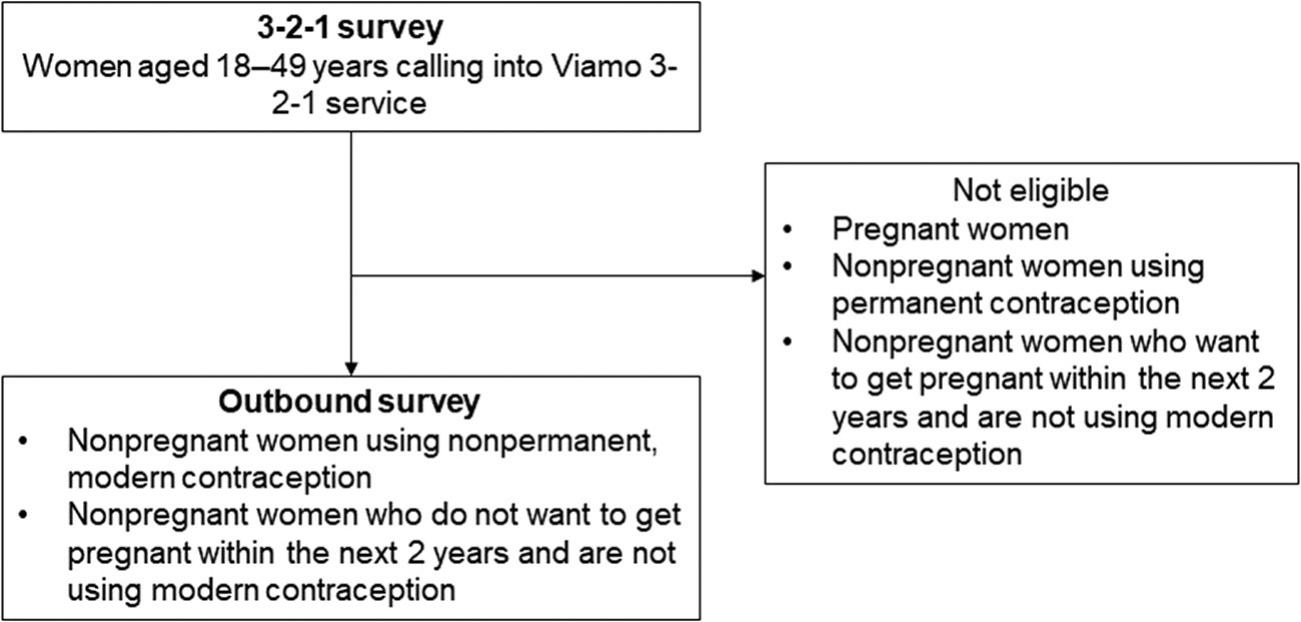
Study Design Summary Examining Behaviors Related to Contraceptive Access and Use During the COVID-19 Pandemic in 4 Countries Abbreviation: COVID-19, coronavirus disease.

### Data Collection and Sample Size

All data collection took place on the phone using interactive voice response. The surveys were available in all languages supported by the 3-2-1 service, including 1 language in Malawi (Chichewa); 1 language in Nepal (Nepali); 5 in Niger (French, Hausa, Zarma, Tamasheq, and Fulfulde); and 6 in Uganda (Luganda, English, Runyakitara, Ateso, Luo, Lugbara). Due to the nature of the platform, the 3-2-1 survey was limited to a maximum of 6 questions per participant; questions confirmed eligibility and covered current pregnancy status and, if applicable, reasons for unintended pregnancies, current contraceptive use, and fertility intentions. The outbound survey documented additional participant characteristics and retrospectively examined contraceptive use and source of supply when the pandemic began in each country. Questions for current modern method users also covered method choice, and, if applicable, reasons for switching methods (current vs. pre-pandemic method) and experiences seeking LARC removal. The survey asked nonusers about their experiences seeking contraceptive methods since the beginning of the pandemic.

We documented consent electronically as a touchpad response by the participant before each survey. There was no compensation for the 3-2-1 survey. For the outbound survey, we randomly selected 10% of participants to receive between US$1–US$2 in phone credit (200 rupees in Nepal, 1,000 Kwacha in Malawi, 1,000 CFA in Niger, and 5,000 shillings in Uganda).

For sample size calculations, we focused primarily on the subpopulation of women who are currently using modern contraception at each round. We calculated sample size to achieve a 95% confidence interval (CI) with 5% precision in the third round of the cohort study to estimate the proportion of women who obtained their preferred method of contraception among current modern contraceptive users who last obtained their method after the beginning of the pandemic. We assumed a conservative estimate of 50% and a 15% loss-to-follow-up rate between consecutive rounds. We aimed for 533 completed surveys per country with this subpopulation in the first outbound survey described in this article, with a plan to continue recruiting women meeting broader eligibility criteria until this target was met.

### Data Analysis

We performed quantitative analyses in Stata version 16 (STATA Corp). Key measures and associated data sources are shown in Table 1. In comparing current and pre-pandemic contraceptive use, we restricted the analysis population to women who responded to the outbound survey to capture changes in behaviors within a consistent sample of women. To examine method switching, we categorized methods as highly effective long-acting (intrauterine devices and implants), effective short-acting (injectables, pills, or lactational amenorrhea method), and less effective short-acting (condoms, emergency contraception, or standard days method). The analysis population for measures related to method choice consists of current modern method users who initiated or resupplied their method after the beginning of the pandemic and is based on last episode of use. Analyses of LARC removals pertain to all current implant and intrauterine contraceptive device (IUD) users, regardless of when they received their method. We examined barriers to access and use among nonusers of modern contraception. We conducted 2 exploratory multivariable logistic regression analyses, separate for each country, to examine changes in modern contraceptive status compared to before the pandemic: adoption (based on current use of nonpermanent, modern contraception) among women who were nonusers pre-pandemic and discontinuation (based on current nonuse of modern contraception) among women who were users pre-pandemic. We included 11 variables related to sociodemographic characteristics and concerns about COVID-19 infection, and, for the discontinuation model only, use of short vs. long-acting method and source of supply when the pandemic began. Variables related to education and income loss were recategorized in some countries due to low sample size for some response options. We confirmed the absence of multicollinearity using variance inflation factor values. We used adjusted odds ratios with their 95% CIs and assessed significance at the 5% level to examine associations based on the logistic models.

**TABLE 1.** Key Outcome Measures and Associated Data Sources

Outcome	Measures	Data Source
Unintended pregnancy	Proportion of pregnant women reporting that their pregnancy was planned at a later time (mistimed) or not planned at all (unplanned)	3-2-1 survey
Contribution of COVID-19 pandemic to unintended pregnancy	Proportion of women with an unintended pregnancy who responded “yes” when asked if the COVID-19 pandemic and the coronavirus social restrictions had affected their ability to avoid or delay getting pregnant	3-2-1 survey
Pre-pandemic modern contraceptive use	Proportion of women who reported they were using an implant, IUD, injectables, pills, emergency contraception, condoms, Standard Days Method, or Lactational Amenorrhea Method when the COVID-19 restrictions began in March 2020 (Malawi, Niger, Uganda) or April/May 2020 (Nepal)	Outbound survey
Current modern contraceptive use	Proportion of women who reported they were using an implant, IUD, injectables, pills, emergency contraception, condoms, Standard Days Method, or Lactational Amenorrhea Method at the time of the survey	3-2-1 survey
Method choice	Proportion of current modern method users who said “yes” when asked if their current method was the method that they wanted to use. The question was only asked of current method users who were using a short-term method (injectables, pills, emergency contraception, condoms, Standard Days Method, or Lactational Amenorrhea Method) and current LARC users who said their method had been inserted after the COVID-19 restrictions began in March 2020 (Malawi, Niger, Uganda) or April/May 2020 (Nepal)	Outbound survey
Barriers to use	Proportion of nonusers of modern contraception who said “yes” when asked if they had wanted to obtain a method since the COVID-19 restrictions began in March 2020 (Malawi, Niger, Uganda) or April/May 2020 (Nepal) and who said “yes” when asked if they had tried to obtain a method	Outbound survey

Abbreviations: COVID-19, coronavirus disease; IUD, intrauterine contraceptive device; LARC, long-acting reversible contraception.

### Ethics Approval

The study was approved by the National Committee on Research in the Social Sciences and Humanities in Malawi, the Ethical Review Board of the Nepal Health Research Council in Nepal, the *Comité National d’Ethique pour la Recherche en Santé* in Niger, the Makerere University School of Public Health Higher Degrees Research and Ethics Committee and the Uganda National Council for Science and Technology in Uganda, and FHI 360’s Protection of Human Subjects Committee in the United States.

## RESULTS

Across countries, 24,809 callers consented to the study and were confirmed eligible (Supplement Figure 1). Among callers found eligible, 92% in Malawi and Uganda (4,936 and 7,378 women, respectively), 89% in Niger (2,602 women), and 79% in Nepal (6,776 women) completed the 3-2-1 survey. Altogether, 12,987 participants in the 3-2-1 survey were eligible for the outbound survey. The response rate for the outbound survey was 53% in Malawi, 36% in Nepal, 41% in Niger, and 33% in Uganda. The mean number of days between the 3-2-1 survey and the outbound survey was 1.5. To assess potential biases due to attrition between the 3-2-1 survey and the outbound survey, we compared the characteristics of eligible women who completed the outbound survey with those of eligible women who did not complete it. The characteristics documented as part of 3-2-1 survey responses were similar between the 2 groups (Supplement Table 1).

### 3-2-1 Survey

The 3-2-1 survey targeted women ages 18–49 years who called into Viamo’s 3-2-1 service. Over 70% of participants in the 3-2-1 survey were aged 18–24 years (Table 2). Between 25% and 38% reported being pregnant. Due to survey timing, all pregnancies happened after the pandemic began.

**TABLE 2.** Characteristics, Contraceptive Use, and Pregnancies Among 3-2-1 Survey Participants

	Malawi, %(N=4,936)	Nepal, %(N=6,776)	Niger, %(N=2,602)	Uganda, %(N=7,378)
Age, years				
18–24	77.9	70.0	79.9	76.7
25–34	21.9	22.8	13.3	19.1
35–49	0.2	7.2	6.8	4.2
Current pregnancy and contraceptive use status^a^				
Pregnant	25.2	25.5	38.3	24.6
Nonpermanent modern method	37.5	29.4	18.1	28.9
Traditional method	0.1	1.0	2.8	2.0
Permanent or any other method	0.2	2.6	7.3	2.7
Nonuser not wanting to get pregnant within 2 years	27.4	29.4	21.9	30.3
Nonuser wanting to get pregnant within 2 years	9.5	12.0	11.7	11.7
Pregnancies by pregnancy intention^b^	n=1,245	n=1,731	n=996	n=1,812
Planned	49.2	69.1	58.7	45.3
Mistimed, not COVID-19-related	5.5	2.4	5.2	4.9
Unplanned, not COVID-19-related	5.9	1.6	5.4	5.9
Mistimed, COVID-19-related	23.8	20.9	17.7	28.4
Unplanned, COVID-19-related	15.7	6.0	13.0	15.5
	n=633	n=535	n=411	n=992
COVID-19-related unintended pregnancies^c^	77.6	87.1	74.2	80.2
Main reason for COVID-19-related unintended pregnancies^d^	n=491	n=466	n=305	n=796
Family planning services closed	22.4	12.9	25.8	19.8
Preferred method unavailable	77.6	87.1	74.2	80.2
Afraid of getting COVID-19	9.0	19.2	5.0	22.8
Family would not allow going to get a method due to COVID-19	18.1	27.6	14.8	20.7
Government restrictions	1.4	0.6	1.6	2.4
Other family planning access reason	4.1	1.1	4.3	2.0
Other reason not related to accessing contraception	34.0	33.5	40.7	44.3

Abbreviations: COVID-19, coronavirus disease; IUD, intrauterine contraceptive device; LAM, lactational amenorrhea method; SDM, standard days method.

a Nonpermanent modern methods include implants, IUD, injectables, pills, emergency contraception, condoms, SDM, and LAM. Traditional methods include withdrawal, rhythm method, or folk methods like herbs.

b Among pregnant women.

c Among pregnant women with an unplanned or mistimed pregnancy.

d Among pregnant women with an unplanned or mistimed pregnancy who reported that COVID-19 affected their ability to avoid or delay pregnancy.

Over half of surveyed pregnant women in Uganda (55%) and Malawi (51%) reported their pregnancy to be unintended, compared to 41% in Niger and 31% in Nepal. Most respondents with unintended pregnancies in our sample (74%–87%) indicated that the pandemic had affected their ability to delay or avoid getting pregnant. Among these, 56%–67% attributed their pregnancy to being unable to access contraception, primarily due to supply-side constraints such as closures (19%–31%) and unavailability of preferred methods (15%–25%), alongside fear of getting infected with COVID-19 (6%–18%).

Between 74% and 87% of respondents with unintended pregnancies at the time of the survey indicated that the COVID-19 pandemic had affected their ability to delay or avoid their pregnancy.

### Outbound Survey

We completed 5,124 outbound surveys (1,694 in Malawi, 1,468 in Nepal, 458 in Niger, and 1,504 in Uganda) with eligible women defined as women in potential need of contraception, including (1) nonpregnant women who reported using nonpermanent modern contraception in the 3-2-1 survey and (2) nonpregnant women who reported they did not want to get pregnant within the next 2 years but were not using modern contraception in the 3-2-1 survey. Altogether, 68%–87% of surveyed respondents to the outbound survey were married and 44%–50% had 2 or more children (Table 3). More respondents were concerned or very concerned about getting infected with COVID-19 in Uganda (77%) and Niger (71%) than in Malawi (51%) and Nepal (41%). Between 73% and 86% reported at least some loss of income in their household since the beginning of the pandemic.

**TABLE 3.** Characteristics of Participants in the Outbound Survey, by Country

	Malawi, %(N=1,694)	Nepal, %(N=1,468)	Niger, %(N=458)	Uganda, %(N=1,504)
Age, years				
18–24	72.7	62.3	79.9	74.5
25–49	27.3	37.7	20.1	25.5
Married, %	73.8	87.3	76.4	68.2
Parity, %				
0	11.7	16.3	25.1	19.4
1	38.1	39.4	27.7	35.0
2	22.7	29.5	20.1	23.3
3+	27.4	14.8	27.1	22.3
Highest education				
None	8.5	12.2	41.9	8.5
Some primary	44.0	33.0	32.8	34.7
Finished primary	22.1	13.3	8.5	27.5
Finished secondary	22.8	22.0	10.7	21.3
More than secondary	2.5	19.6	6.1	8.0
Concern about getting infected with COVID-19				
A little/not concerned or has/had COVID-19	49.1	58.8	28.6	22.5
Concerned	19.3	18.3	28.8	23.9
Very concerned	31.6	23.0	42.6	53.5
Household income loss during COVID-19				
None	16.3	19.4	26.9	13.9
Small part	56.6	33.8	53.3	42.6
Moderate part	9.0	19.2	5.0	22.8
Large part/all	18.1	27.6	14.8	20.7

Abbreviation: COVID-19, coronavirus disease.

### Modern Contraceptive Use and Source of Supply

We compared contraceptive behaviors before the start of the pandemic to the time of the survey in each country among respondents who completed the outbound survey. Respondents were women who were current users of nonpermanent, modern contraception or who did not want to get pregnant within 2 years but were not using a modern method at the time of the survey. The proportion of these women in our sample who had been using a method before the pandemic was 10% higher compared to the time of the survey in Malawi and Uganda and 7% higher in Nepal (Table 4). In Niger, it was 2% lower. Before the pandemic, 41%–67% of nonpermanent, modern contraceptive surveyed users were using implants or IUDs, compared to 37%–50% at the time of the survey. The proportion of LARC users decreased across all countries, dropping from between 5% in Nepal to 22% in Malawi.

**TABLE 4.** Contraceptive Use Dynamics Among Outbound Survey Participants,^a^ by Country

	Malawi, %(N=1,694)	Nepal, %(N=1,468)	Niger, %(N=458)	Uganda, %(N=1,504)
Pre-pandemic contraceptive use				
Pre-pandemic contraceptive use, including traditional methods	78.4	57.4	46.5	66.4
Pre-pandemic modern contraceptive use	75.0	54.7	40.4	59.8
Pre-pandemic modern method mix^b^	n=1,271	n=803	n=185	n=900
Implant	54.8	32.8	48.1	36.9
IUD	8.3	8.6	18.9	15.0
Injectable	22.3	23.0	13.5	21.7
Pill	4.3	2.1	13.5	4.1
Emergency contraception	2.1	10.3	2.7	4.0
Condoms	7.8	22.8	0.0	14.7
SDM/cycle beads or LAM	0.4	0.4	3.2	3.7
Pre-pandemic source of supply^c^	n=1,268	n=800	n=182	n=887
Public sector facility	83.5	76.6	65.9	65.5
Private sector facility	8.6	15.8	15.4	20.3
Community health worker or outreach event	6.7	3.3	11.0	8.0
Pharmacy, chemical, or drug shop	1.2	4.1	4.9	5.2
Ordered on a website, app, or phone	0.0	0.3	2.7	1.0
Current contraceptive use				
Current contraceptive use, including traditional methods	58.1	50.7	49.6	53.9
Current modern contraceptive use	58.0	48.2	42.1	48.8
Current modern method mix^d^	n=982	n=708	n=193	n=734
Implant	44.5	28.4	33.7	32.8
IUD	5.9	8.5	10.9	10.6
Injectable	30.2	25.1	21.2	23.7
Pill	3.4	11.2	23.3	6.7
Emergency contraception	4.3	3.1	5.7	3.4
Condoms	8.8	23.2	1.0	17.3
SDM/cycle beads or LAM	3.0	0.6	4.1	5.4
Current source of supply^e^	n=622	n=573	n=159	n=592
Public sector facility	77.8	74.5	62.3	66.2
Private sector facility	10.5	14.8	15.1	21.8
Community health worker or outreach event	9.8	3.5	12.6	6.1
Pharmacy, chemical, or drug shop	1.4	6.8	2.5	4.6
Ordered on a website, app, or phone	0.5	0.3	7.5	1.4
Main reason for choosing current source of supply^e^	n=622	n=573	n=159	n=592
Family planning services closed elsewhere	87.5	26.9	29.6	21.6
Preferred method unavailable elsewhere	11.7	23.9	28.9	18.8
Afraid of getting COVID-19 elsewhere	0.3	25.1	18.9	45.8
Family would not allow going elsewhere due to COVID-19	0.3	3.7	6.9	4.7
Government restrictions	0.2	5.8	9.4	7.4
Other	0.0	14.7	6.3	1.7
Changes in contraceptive use				
Change in modern contraceptive status	n=1,694	n=1,468	n=458	n=1,504
Consistent user	50.0	40.1	23.4	39.6
Discontinuer	25.0	14.6	17.0	20.2
Adopter	8.0	8.1	18.8	9.2
Consistent nonuser	17.0	37.2	40.8	31.0
Change in contraceptive methods^f^	n=847	n=589	n=107	n=596
Less effective method	18.2	11.2	30.8	16.6
Method as effective	77.1	74.5	61.7	77.3
More effective method	4.7	14.3	7.5	6.0

Abbreviations: COVID-19, coronavirus disease; EC, emergency contraception; IUD, intrauterine device; LAM, lactational amenorrhea method; SDM, standard days method.

a Outbound survey participants include (1) nonpregnant women using nonpermanent, modern contraception and (2) nonpregnant women who do not want to get pregnant within the next two years and are not using modern contraception. Due to study design, in this table, modern methods refer to nonpermanent, modern contraceptive methods including implants, IUDs, injectables, pills, emergency contraception, condoms, SDM, and LAM.

b Among pre-pandemic modern method users.

c Among pre-pandemic users of modern contraception other than LAM.

d Among current users of modern, nonpermanent contraception (implants, IUDs, injectables,, pills, EC, condoms, SDM, or LAM).

e Among current users of modern contraception other than LAM who obtained/resupplied during COVID-19.

f Among consistent users of modern contraception.

Most surveyed nonpermanent, modern method users reported sourcing their method from public sector facilities before the pandemic (66%–84%) and at the time of the survey (62%–78%) (Table 4). In Uganda, more surveyed women who obtained their method through community health workers or outreach programs before the pandemic discontinued modern contraception compared to other sources of supply. In Nepal and Niger, discontinuation was higher among respondents who obtained their methods in private clinics before COVID-19 (Supplement Table 2). The most common source of supply among surveyed adopters was public facilities (Supplement Table 3). Between 16% and 35% of consistent users switched types of sources of supply. More consistent contraceptive users who obtained their method from private health facilities or community or online sources before the pandemic switched to another source (primarily public sector facilities), compared to public facility clients (Supplement Table 4).

In Uganda, 46% of surveyed nonpermanent modern method users cited fear of being infected with COVID-19 as the main reason for choosing their current source of supply, compared to 25% in Nepal and 19% in Niger (Table 4). Almost all Malawian surveyed users mentioned supply-side factors (99%), compared to 40%–59% of women in other countries.

Large proportions of surveyed women in potential need of contraception had the same contraceptive status of use/nonuse at the 2 time points (pre-pandemic and at time of survey), ranging between 64% in Niger to 77% in Nepal. In Malawi, Nepal, and Uganda, 40%–50% of surveyed women in potential need of contraception remained users (consistent users) while 17%–37% remained nonusers (consistent nonusers). In Niger, fewer respondents remained users than nonusers (23% vs. 41%). More respondents discontinued (discontinuers) than adopted (adopters) a modern method in Malawi (25% vs. 8%), Uganda (20% vs. 9%), and Nepal (15% vs. 8%), whereas in Niger, slightly more respondents adopted than discontinued a method (19% vs. 17%). Most respondents who were consistent users kept the same method or switched to a method as effective as the one they were previously using, ranging from 62% in Niger to 77% in Malawi and Uganda. More respondents reported switching to less effective methods than to more effective methods in Malawi (18% vs. 5%), Niger (32% vs. 8%), and Uganda (16% vs. 6%). In Nepal, more respondents switched to more effective methods than to less effective methods (14% vs. 11%).

In the multivariable models for surveyed women in potential need of contraception who were not using a nonpermanent, modern method before the pandemic (adopters vs. consistent nonusers), married women in Malawi, younger women and married women in Nepal, and women with 2 or more children in Niger had higher odds of currently using a method. Women who reported little or no concern about COVID-19 in Niger also had higher odds of uptake compared to those reporting they were concerned or very concerned (Table 5). Among surveyed women in potential need of contraception who were using a nonpermanent, modern method before the pandemic (discontinuers vs. consistent users), women with higher education and women who were very concerned about getting infected with COVID-19 had higher odds of discontinuation in Malawi, as well as women with lower education in Niger and women who obtained their method before the pandemic from community sources in Uganda. Other associations were not found to be statistically significant in this analysis.

**TABLE 5:** Adjusted Odds Ratio Estimates and 95% Confidence Intervals for Factors Associated With Adoption and Discontinuation of Modern Contraception During the COVID-19 Pandemic, by Country

	Malawi		Nepal		Niger		Uganda	
Factor	Adoption AmongNonusers,^a^ AOR(95% CI)(n=423)	DiscontinuationAmong Users, AOR(95% CI)(n=1,268)	Adoption AmongNonusers,^a^ **AOR**(95% CI)(n=665)	DiscontinuationAmong Users, AOR(95% CI)(n=800)	Adoption AmongNonusers,^a^ **AOR**(95% CI)(n=273)	DiscontinuationAmong Users, AOR(95% CI)(n=182)	Adoption AmongNonusers,^a^ **AOR**(95% CI)(n=604)	DiscontinuationAmong Users, AOR(95% CI)(n=887)
Age (25–49 vs. 18–24^b^)	0.80 (0.47, 1.35)	0.74 (0.55, 1.00)	0.51 (0.31, 0.84)^c^	0.76 (0.53, 1.10)	1.12 (0.57, 2.18)	0.66 (0.29, 1.47)	0.91 (0.56, 1.49)	1.10 (0.79, 1.53)
Married	2.23 (1.40, 3.54)^c^	0.70 (0.52, 0.93)	3.06 (1.53, 6.10)^c^	0.73 (0.41, 1.29)	1.74 (0.93, 3.25)	0.94 (0.40, 2.21)	1.02 (0.67, 1.54)	0.87 (0.63, 1.18)
Parity (2+ vs. 0–1^b^)	1.13 (0.69, 1.84)	0.97 (0.74, 1.27)	1.36 (0.87, 2.13)	0.86 (0.61, 1.23)	1.31 (0.75, 2.27)	0.82 (0.42, 1.60)	2.00 (1.31, 3.06)^c^	1.10 (0.81, 1.49)
Education (higher vs. lower^b^^,d^)	0.98 (0.62, 1.53)	1.34 (1.04, 1.71)^c^	0.71 (0.46, 1.10)	0.92 (0.66, 1.28)	1.22 (0.71, 2.10)	0.44 (0.24, 0.84)^c^	0.79 (0.53, 1.18)	0.89 (0.66, 1.19)
Concern about COVID-19 infection (little/not concerned or has/had COVID-19^b^*)*								
Concerned	1.18 (0.67, 2.07)	0.86 (0.62, 1.20)	0.76 (0.41, 1.42)	1.00 (0.66, 1.51)	0.55 (0.27, 1.10)	1.10 (0.49, 2.51)	0.46 (0.26, 0.81)^c^	0.74 (0.49, 1.14)
Very concerned	0.85 (0.52, 1.40)	1.34 (1.02, 1.75)^c^	1.47 (0.91, 2.37)	0.92 (0.61, 1.37)	0.93 (0.51, 1.69)	0.60 (0.27, 1.33)	0.60 (0.38, 0.96)^c^	0.93 (0.65, 1.33)
Household income loss due to COVID-19 (none^b^*)*								
Small	n/a	n/a	1.80 (0.98, 3.30)	0.97 (0.62, 1.52)	n/a	n/a	1.39 (0.75, 2.58)	0.83 (0.53, 1.30)
Moderate/large/all	n/a	n/a	1.51 (0.82, 2.76)	0.90 (0.58, 1.40)	n/a	n/a	1.32 (0.72, 2.44)	1.09 (0.70, 1.70)
Any	0.96 (0.53, 1.74)	1.37 (0.97, 1.91)	n/a	n/a	1.09 (0.57, 2.09)	0.86 (0.42, 1.74)	n/a	n/a
Pre-pandemic method (long-acting vs. short-acting^b^*)*	n/a	0.85 (0.66, 1.09)	n/a	0.89 (0.64, 1.25)	n/a	0.66 (0.34, 1.27)	n/a	0.84 (0.63, 1.13)
Pre-pandemic source of supply (public facility^b^)								
Private facility	n/a	1.02 (0.67, 1.56)	n/a	1.49 (0.98, 2.27)	n/a	1.31 (0.54, 3.21)	n/a	1.35 (0.94, 1.94)
CHW/outreach/pharmacy/online	n/a	0.89 (0.57, 1.40)	n/a	0.89 (0.47, 1.68)	n/a	1.18 (0.52, 2.66)	n/a	2.08 (1.38, 3.12)^c^

Abbreviations: AOR, adjusted odds ratio; CHW, community health worker; CI, confidence interval; COVID-19, coronavirus disease.

a Nonusers are nonpregnant women who reported using a traditional method or not using any form of contraception, and also reported that they did not want to get pregnant in the next 2 years. Users include current users of nonpermanent, modern contraception.

b Reference.

c Statistically significant (*P* ≤ .05).

d In Malawi, Nepal and Uganda, lower includes none and some primary, and higher includes finished primary, finished secondary, and higher. In Niger, lower includes none, and higher includes all other categories.

### Method Choice

In our sample, 83.9% (95% CI=80.8, 86.7) of current nonpermanent, modern method users who last obtained or resupplied their method after the beginning of the pandemic in Malawi, 87.8% (95% CI=84.9, 90.4) in Nepal, 79.4% (95% CI=72.4, 85.3) in Niger, and 84.2% (95% CI=81.0, 87.0) reported they were using their preferred method (Table 6). Of surveyed nonpermanent, modern method users who reported they did not obtain their preferred method, the most commonly reported barriers related to supply, including the method not being available (31%–52%) and providers being unable to provide the preferred method (16%–46%). Regarding demand, 22% of Ugandan respondents, 6% of Malawian respondents, and 5% of Nepali respondents cited fear of infection with COVID-19, while 15% of respondents in Uganda, 12% in Malawi, 8% in Niger, and 7% in Nepal said they did not have enough money.

**TABLE 6.** Method Choice^a^ Among Current Nonpermanent Method Users and LARC Removals Among LARC Users, by Country

	Malawi	Nepal	Niger	Uganda
	n=635	n=575	n=165	n=606
Obtained preferred method, % (95% CI)	83.9 (80.8, 86.7)	87.8 (84.9, 90.4)	79.4 (72.4, 85.3)	84.2 (81.0, 87.0)
Main reason for not obtaining preferred method, %^b^	n=78	n=44	n=26	n=65
Preferred method unavailable	34.6	52.3	30.8	30.8
Provider unable to provide preferred method	20.5	15.9	46.2	24.6
Provider recommended another method/not eligible	17.9	20.5	15.4	4.6
Not enough money	11.5	6.8	7.7	15.4
Afraid of getting COVID-19	6.4	4.5	0.0	21.5
Other	9.0	0.0	0.0	3.1
LARC removals^c^	n=495	n=261	n=86	n=319
Tried to get LARC removed, %	45.7	36.8	54.7	17.6
Main reason for not being able to have LARC removed, %^d^	n=226	n=96	n=47	n=56
No provider available/place closed	35.8	67.7	46.8	21.4
No equipment/supplies for removal	23.0	10.4	21.3	25.0
Provider advised to keep method	20.8	9.4	10.6	23.2
Not enough money	11.1	7.3	12.8	21.4
Other	9.3	5.2	8.5	8.9

Abbreviations: CI, confidence interval; COVID-19, coronavirus disease; IUD, intrauterine device; LAM, lactational amenorrhea method; LARC, long-acting reversible contraception.

a At last episode of use: calculated for current modern method users who last obtained/resupplied their method during COVID-19 or who use LAM.

b Among current modern method users who last obtained their method during COVID-19 and are not using their preferred method.

c Among current implant and IUD users.

d Among current implant and IUD users who tried to have their method removed.

Overall, 55% of respondents using an implant or IUD at the time of the survey in Niger, 46% in Malawi, and 37% in Nepal said they had tried unsuccessfully to get their method removed since the beginning of the pandemic, compared to 18% of LARC users in Uganda. The main barriers to getting a removal in Malawi and Niger were no provider (36%–47%) and lack of equipment (21%–23%), with 21% of Malawian respondents also indicating the provider advised them to keep their method. In Nepal, 68% of respondents mentioned no provider as the main barrier.

### Barriers to Use

Among surveyed women who did not want to get pregnant within 2 years but were not using a modern method at the time of the survey, 52%–53% in Malawi and Uganda, 37% in Niger, and 29% in Nepal had tried to get a method since the beginning of the pandemic; an additional 9%–14% had wanted to use a method but had not tried to obtain one (Table 7). Altogether, 60% of Nepali respondents and about a third (29%–33%) of respondents in other countries who tried obtaining a method said their preferred method was unavailable. In addition, 22%–26% of respondents in Niger and Malawi indicated that the provider was not able to provide their preferred method, while 31%–32% of respondents in Uganda and Niger said they did not have enough money. Around half of the surveyed women who wanted a method but did not try to obtain one reported being afraid of being infected with COVID-19 (49%–52%), while 17%–21% mentioned service closures.

**TABLE 7.** Barriers to Use Among Current Nonusers^a^ of Modern Contraception, by Country

	Malawi, %	Nepal, %	Niger, %	Uganda, %
	n=712	n=760	n=265	n=770
Intention to get modern method				
Did not want method	34.1	62.1	50.9	34.4
Wanted method, did not try obtaining one	13.6	9.3	12.1	12.5
Tried obtaining a method	52.2	28.6	37.0	53.1
Reasons for not trying to obtain a method^b^	n=97	n=71	n=32	n=96
Family planning services closed	20.6	21.1	18.8	16.7
Afraid of getting COVID-19	48.5	52.1	50.0	49.0
Family would not allow going to get a method due to COVID-19	2.1	7.0	12.5	12.5
Government restrictions	15.5	7.0	9.4	9.4
Other	13.4	12.7	9.4	12.5
Reasons for not obtaining a method among those who tried^c^	n=372	n=217	n=98	n=409
Preferred method unavailable	31.2	60.4	32.7	29.3
Provider unable to provide preferred method	25.8	12.9	22.4	13.0
Provider recommended another method	11.8	8.8	10.2	15.6
Not enough money	12.4	7.4	31.6	30.8
Other	18.8	10.6	3.1	11.2

Abbreviation: COVID-19, coronavirus disease.

a Nonusers include nonpregnant women who reported using a traditional method or not using any form of contraception but did not want to get pregnant in the next two years.

b Among current nonusers of modern contraception who wanted a method but did not try obtaining one.

c Among current nonusers of modern contraception who tried obtaining a method.

## DISCUSSION

This assessment conducted approximately 1 year into the pandemic in 4 countries was intended to shed some light on women’s experiences accessing and using contraception under COVID-19. The study used a convenience sample of users of Viamo’s 3-2-1 service to rapidly identify women in potential need of contraceptive services and reach them safely while avoiding personal contact. This assessment was not designed to be nationally representative or to produce population-level estimates of the effects of the pandemic on unintended pregnancies and FP behaviors. Rather, given the paucity of information available from the perspective of women at the time, we aimed to provide timely evidence to program managers and to improve their understanding of the potential ways women’s contraceptive journey may indirectly be affected by the pandemic. For similar reasons, analysis results were not intended to be compared across countries. Nonetheless, presenting results from 4 countries is valuable to illustrate the FP service experiences of surveyed women under COVID-19 in different contexts.

We aimed to provide timely evidence to program managers and to improve their understanding of the potential ways women’s contraceptive journey may indirectly be affected by the pandemic.

In all 4 countries, the assessment sample skewed toward young women, with 70% or more of participants in the 3-2-1 survey and 62% or more of participants in the outbound survey being aged 18–24 years. In contrast, available estimates from national surveys report 42% of women aged 15–24 years among women of reproductive age in Malawi, 38% in Nepal, 34% in Niger, and 44% in Uganda.^8^^,^^9^^,^^16^^,^^17^ Therefore, our results primarily illustrate the potential vulnerabilities of younger women under COVID-19. In the 3-2-1 survey, we found evidence of participants attributing unintended pregnancies to the pandemic. To gain more detailed insight into experiences accessing and using contraception since the beginning of the pandemic, the outbound survey focused on women who were modern, nonpermanent method users and those who did not want to get pregnant within 2 years at the time of the survey but were not using a modern method. Within that group, the proportion of respondents to our survey who reported using a modern contraceptive method decreased compared to before the pandemic in all countries except Niger. By design, all participants in the outbound survey were in potential need of contraception. While we did not assess fertility intentions before the pandemic, if we assume similar intentions before the pandemic, we argue, based on study results that the ability to realize their contraceptive intentions has gotten worse overall for the women reached by our study in Malawi, Nepal, and Uganda. Even if fewer women in our sample were in need of contraception before the pandemic, we argue that the contraceptive outcomes of the women who participated in our study would similarly be worse overall. This is because at least some of the nonusers before the pandemic (those not in need of contraception at that time) could be considered to have fulfilled their contraceptive intentions before the pandemic, leading to an even higher proportion of respondents realizing their contraceptive intentions before the pandemic while there would be no change at the time of the survey. In Niger, where fairly similar proportions of surveyed women reported modern contraceptive use before and during the pandemic, we note that there were no pandemic-related temporary health services closures during the data collection period.

Because our findings only capture the experiences of women reached by our study, continuing to generate evidence on the experiences of women from different settings over time remains important to monitor potential implications of the pandemic on access to essential FP services and to inform adjustments to programming and policy. Examining the experiences of different population subgroups is also critical to unmask potential vulnerabilities, as supported by PMA analyses showing differential impacts of the pandemic for some sociodemographic groups, including young women, in some settings.^7^

Our assessment examined respondents’ perspectives on the contributions of supply factors, demand factors, and government restrictions to the challenges experienced with contraceptive access and use. Findings point at the women in our study experiencing notable supply and demand-side constraints across countries. First, temporary service closures constrained choice of source of supply, especially in Malawi. Results also linked closures to unintended pregnancies and nonuse.

Second, product shortages affected the source of supply and method choice among surveyed current users, as well as method uptake among nonusers (women who did not want to get pregnant within 2 years but were not using modern contraception). Notably, while some respondents chose other contraceptive methods, our findings linking unavailability of one’s preferred method to nonuse and unintended pregnancies indicate that elasticity of demand is limited. Method choice is an essential tenet of FP services. Our results affirm the deleterious effects of the unavailability of a wide range of methods: respondents who managed to overcome other potential access barriers walked away with no method after making contact with services despite the pandemic. While supply chain management is a chronic challenge in many settings, additional disruptions have arisen during the pandemic.^2^^,^^18^ Strategies including prepositioning commodities, identifying alternative suppliers, and more broadly improving product flow and data management for quantification and requisitioning should be considered for more resilient supply chains.^5^^,^^19^^,^^20^ If a woman’s preferred method is not available, counseling on other contraceptive options should be readily available.^21^

Our results affirm the deleterious effects of the unavailability of a wide range of methods: respondents who managed to overcome other access barriers walked away with no method after making contact with services.

Third, fear of COVID-19 infection was the leading reason for not trying to obtain a method among the nonusers reached by this study and a cited cause for unintended pregnancies. In multivariable models, it was significantly associated with discontinuation among users in Malawi and with not adopting a method among nonusers in Niger. This is consistent with evidence that public fear of infection and lack of confidence in health services can reduce service utilization during epidemics, and with COVID-19-specific findings from Burkina Faso and Kenya where it was similarly evoked as a reason for nonuse.^4^^,^^22^^,^^23^ In our assessment, fear of contracting COVID-19 also influenced method choice and source of supply, especially in Uganda, where levels of concern about getting infected were highest among study participants. These findings point to the need to integrate guidance on how to access care for reasons not related to COVID-19 into public health messaging on infection control, including information on prevention measures within health facilities, and to ensure enough supplies are available for these measures to be adhered to. Multimonth refills of contraceptive methods should also be offered to clients for extended periods and possibly expanded to more service delivery points like drug shops and pharmacies to minimize trips and optimize access.^21^^,^^24^ For example, Nepal, Niger, and Uganda included multimonth supply of oral contraceptive pills in their guidance documents.^25^^–^^27^ Additionally, countries should consider waiving or decreasing prescription costs to overcome financial barriers that may result from income loss during COVID-19.^28^

Global COVID-19 guidance recommends alternative service delivery models to mitigate service disruptions, including integrated, community-based, and digital approaches—notably through the application of High Impact Practices in Family Planning.^19^^,^^21^ Despite supply-side constraints and fear of infections, public sector facilities remained the main source of supply during COVID-19 for the women in our assessment. A larger share of respondents who were public facility clients discontinued modern method use compared to respondents who were users of other sources; however, public health facilities proportionately retained a larger share of their clients among the women who were consistent users in this assessment and were the main recipient of clients switching from other sources of supply. In Uganda, community services were temporarily suspended, so some clients may have shifted to public sector facilities. While this finding may be somewhat surprising, it may be partially explained by the fact that data collection spanned several months during which restrictions fluctuated or by the nature of our sample. More longitudinal research is needed to understand how patterns of service utilization may fluctuate during more or less acute phases of the pandemic (e.g., lockdowns or COVID-19 wave peaks) or compare them to those observed in normal times.

For women using implants or IUDs, removals are an important facet of informed choice. Ensuring access to removals at the time of women’s choosing is a topic that is receiving increasing attention, notably given the rapid rise of implant use in sub-Saharan Africa.^29^^,^^30^ Conversely, extended use of LARCs beyond their labeled duration is effective, and postponing removals with proper counseling can be an appropriate strategy when service disruptions are encountered.^31^^,^^32^ At the same time, several of the factors reported as reasons for not obtaining a removal in this assessment, including provider unavailability, lack of equipment, or being counseled to keep the method, have been reported as barriers to accessing removals in normal times.^30^^,^^33^ More research is needed to establish whether these factors are systemic barriers or related to the pandemic.

### Strengths and Limitations

This 4-country assessment offers an examination of several dimensions of contraceptive access and use and allows for a richer understanding of the ways that the COVID-19 pandemic may have indirectly affected women’s contraceptive journey. The use of interactive voice response and the recruitment approach through an established, popular phone-based service provide a useful strategy to gather valuable information on women’s experiences under emergency conditions and pinpoint potential areas in need of strengthening to continue serving contraceptive needs.

However, findings must be interpreted while considering self-selection and selection bias. Results are only applicable to users of the Viamo 3-2-1 service who opted into the survey. Mobile subscriber penetration is 38% in Malawi, 63% in Nepal, 38% in Niger, and 51% in Uganda.^34^ We also noted earlier that the proportion of women ages 18–24 years in this assessment is high. We experienced sizable attrition between the 3-2-1 and outbound surveys. Comparisons are difficult because this assessment was the first instance of Viamo combining a survey on its 3-2-1 platform with an outbound survey; however, the typical completion rate Viamo has encountered on stand-alone outbound surveys is considerably lower than the ones in this assessment (about 10%).^35^^–^^37^ Although our comparison of eligible women who completed the outbound survey to those who did not complete it did not reveal major differences between the limited number of variables collected in the 3-2-1 survey, unmeasured differences cannot be ruled out. The approximate characterization of women in need of contraception informing sample selection for the outbound survey is based on contraceptive use and fertility intentions measured 1 year into the pandemic; sample selection and analyses do not account for changes in need of contraception from before the start of the pandemic. Due to these limitations, estimates such as modern contraceptive use should not be directly compared to pre-pandemic national estimates for women of reproductive age. We only examined adoption and discontinuation based on a comparison of nonpermanent modern contraceptive use pre-pandemic and at the time of the survey. Thus, those who have switched to permanent methods were not examined in this study. In addition, our results do not necessarily imply continued use/nonuse between these 2 time points for women categorized as consistent users or nonusers. Survey questions were administered using interactive voice response, with limited response options. Due to the rapidly evolving nature of the pandemic, the data collection period extending over several weeks allowed for shifts in the epidemiological context and response measures, complicating the interpretation of some findings. Pre-pandemic data were obtained retrospectively. While the beginning of the pandemic may be considered a memorable event and facilitate recollection, the possibility of recall bias exists. Lastly, the design does not systematically allow differentiation between COVID-19-related challenges and preexisting weaknesses of health systems.

## CONCLUSION

Our assessment conducted 1 year into the pandemic in Malawi, Nepal, Niger, and Uganda brings much-needed insights into women’s experiences with contraceptive access and use during the COVID-19 pandemic. We found evidence of participants reached by this study attributing unintended pregnancies to pandemic-related access constraints across countries. Fewer surveyed women in potential need of contraception reported current use of modern contraception compared to before the pandemic in 3 of 4 countries. There were also missed opportunities among nonusers who were afraid to seek services or who accessed services but left without a method. The effects of the pandemic must be interpreted within the contraceptive, health system, and epidemiological context of each country, and longitudinal data are needed as patterns may change over time. Our results provided the basis for fruitful discussion in the 4 countries given the overall paucity of data on women’s experiences and country commitments to ensuring continuity of essential services through the pandemic (Box). These data provide a useful complementary perspective to what health monitoring information systems can capture, can inform program adjustments, and help countries plan for future crises.

BOX **Key Recommendations Produced by Stakeholders in 4 Countries**

**Malawi**

- Address hesitancy to access health services at health facilities through social and behavioral change communication.
- Strengthen the supply chain management system to improve commodity availability at all levels.
- Coordinate health worker shifts within health facilities to ensure consistent availability of services.

**Nepal**

- Improve availability of the full method mix through the public and private sector.
- Increase access to contraceptive methods through total market approach.
- Address demand-side barriers to access.

**Niger**

- Strengthen the provision of quality services through community-based distribution of contraceptives including injectables.
- Expand mobile outreach.
- Support integration of family planning in other health services (e.g., postpartum care, malaria, and nutrition).

**Uganda**

- Encourage vaccination of health providers, which increases women’s confidence to access services.
- Ensure affordability and a full method mix at public facilities.
- Engage private sector for continuity of services.

## Supplementary Material

GHSP-D-22-00063-supplement.docx

## Notes

**Peer Reviewed**
