# Supplementary material for: Women’s Experiences With Family Planning Under COVID-19: A Cross-Sectional, Interactive Voice Response Survey in Malawi, Nepal, Niger, and Uganda
Source: Glob Health Sci Pract. 2022 Aug 30;10(4):e2200063. doi: 10.9745/GHSP-D-22-00063 (PMC9426982; doi:10.9745/GHSP-D-22-00063)
Supplement: GHSP-D-22-00063-supplement.docx [file GHSP-D-22-00063-supplement.docx]

**Supplement Figure 1.** Flow Chart for Malawi, Nepal, Niger, and Uganda


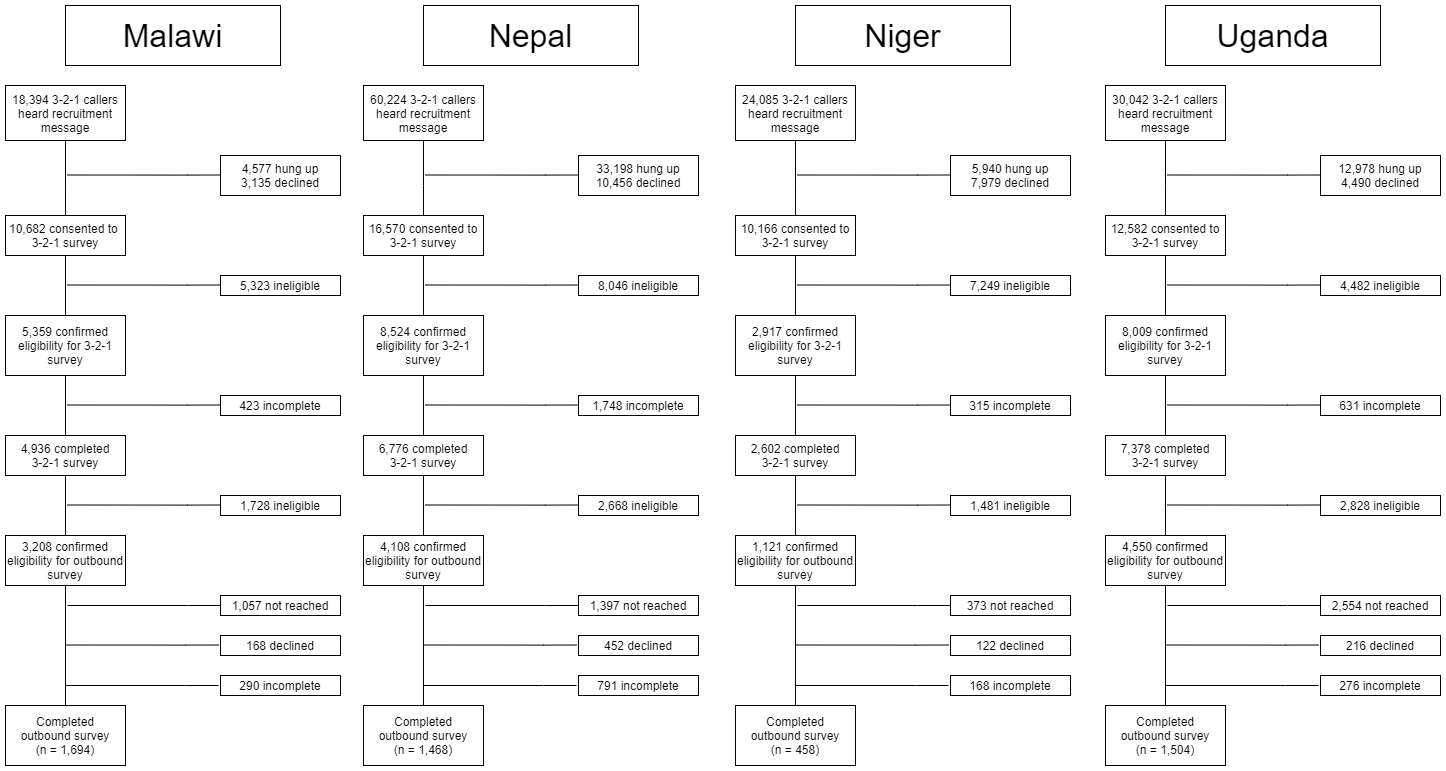


**Supplement Table 1.** Comparison of Characteristics of Women Eligible for the Outbound Survey Who Did and Did Not Complete the Survey

|  | Malawi | | Nepal | | Niger | | Uganda | |
| --- | --- | --- | --- | --- | --- | --- | --- | --- |
|  | Completers | Non-completers | Completers | Non-completers | Completers | Non-completers | Completers | Non-completers |
|  | n = 1694 | n = 1514 | n = 1468 | n = 2640 | n = 458 | n = 663 | n = 1504 | n = 3046 |
| Age, years, % |  |  |  |  |  |  |  |  |
| 18-24 | 72.7 | 77.2 | 62.3 | 65.8 | 79.9 | 81.5 | 74.5 | 76.8 |
| 25-34 | 27.0 | 22.7 | 29.3 | 25.5 | 13.5 | 12.8 | 21.7 | 18.6 |
| 35-49 | 0.3 | 0.1 | 8.4 | 8.7 | 6.6 | 5.7 | 3.8 | 4.6 |
| Eligibility subgroup, % |  |  |  |  |  |  |  |  |
| Non-pregnant women using non-permanent, modern contraception | 42.0 | 42.5 | 51.8 | 51.3 | 57.9 | 57.9 | 51.2 | 54.2 |
| Non-pregnant women who do not want to get pregnant within the next 2 years but are not using modern contraception | 58.0 | 57.5 | 48.2 | 48.7 | 42.1 | 42.1 | 48.8 | 45.8 |
| Current method used among users, % | n = 982 | n = 871 | n = 708 | n = 1286 | n = 193 | n = 279 | n = 734 | n = 1395 |
| Implant | 44.5 | 38.1 | 28.4 | 24.4 | 33.7 | 29.8 | 32.8 | 29.2 |
| IUD | 5.9 | 7.8 | 8.5 | 11.2 | 10.9 | 16.9 | 10.6 | 10.3 |
| Injectable | 30.2 | 29.9 | 25.1 | 23.5 | 21.2 | 24.7 | 23.7 | 21.7 |
| Pill | 3.4 | 4.3 | 11.2 | 12.7 | 23.3 | 16.1 | 6.7 | 8.6 |
| Emergency contraception | 4.3 | 4.5 | 3.1 | 3.0 | 5.7 | 3.6 | 3.4 | 3.5 |
| Condoms | 8.8 | 10.1 | 23.2 | 23.7 | 1.0 | 2.2 | 17.3 | 20.2 |
| SDM/Cycle beads or LAM | 3.0 | 5.4 | 0.6 | 1.6 | 4.2 | 6.8 | 5.5 | 6.5 |

**Supplement Table 2.** Proportion of Pre-Covid Users Who Discontinued Modern Contraception Other Than LAM by Pre-COVID Source of Supply, by Country

|  | Malawi | Nepal | Niger | Uganda |
| --- | --- | --- | --- | --- |
|  | n = 1059 | n = 613 | n = 120 | n = 581 |
| Public sector facility, % | 33.1 | 25.3 | 39.2 | 29.8 |
|  | n = 109 | n = 126 | n = 28 | n = 180 |
| Private sector facility, % | 34.9 | 34.1 | 53.6 | 36.7 |
|  | n = 85 | n = 26 | n = 20 | n = 71 |
| Community health worker or outreach event, % | 30.6 | 30.8 | 45.0 | 50.7 |
|  | n = 15 | n = 33 | n = 9 | n = 46 |
| Pharmacy, chemical, or drug shop, % | 53.3 | 18.2 | 33.3 | 39.1 |
|  | n = 0 | n = 2 | n = 5 | n = 9 |
| Ordered on a website, app, or phone, % | 0.0 | 0.0 | 60.0 | 66.7 |

Community or online source includes community health worker, outreach event, pharmacy, chemical, or drug shop and ordering on a website, app, or phone

**Supplement Table 3.** Current Source of Supply Among Adopters of Modern Contraception Other Than LAM, by Country

|  | Malawi | Nepal | Niger | Uganda |
| --- | --- | --- | --- | --- |
|  | n = 130 | n = 117 | n = 83 | n = 130 |
| Public sector facility, % | 73.1 | 68.4 | 45.8 | 56.9 |
| Private sector facility, % | 12.3 | 14.5 | 21.7 | 24.6 |
| Community health worker or outreach event, % | 12.3 | 5.1 | 18.1 | 8.5 |
| Pharmacy, chemical, or drug shop, % | 1.5 | 10.3 | 2.4 | 5.4 |
| Ordered on a website, app, or phone, % | 0.8 | 1.7 | 12.0 | 4.6 |

Community or online source includes community health worker, outreach event, pharmacy, chemical, or drug shop and ordering on a website, app, or phone.

**Supplement Table 4.** Changes in Source of Supply Among Consistent Users of Modern Contraception, by Country

|  | Malawi | Nepal | Niger | Uganda |
| --- | --- | --- | --- | --- |
|  | n = 491 | n = 455 | n = 75 | n = 456 |
| Switched source of supply, % | 17.9 | 16.0 | 34.7 | 19.3 |
| Change in source of supply among public facility clients, % | n = 384 | n = 340 | n = 51 | n = 296 |
| Did not change source | 90.9 | 92.6 | 88.2 | 91.6 |
| Switched to private facility | 4.4 | 4.1 | 7.8 | 6.1 |
| Switched to community or online source | 4.7 | 3.2 | 3.9 | 2.4 |
| Change in source of supply among private facility clients, % | n = 54 | n = 73 | n = 12 | n = 100 |
| Did not change source | 51.9 | 63.0 | 8.3 | 71.0 |
| Switched to public facility | 33.3 | 26.0 | 75.0 | 20.0 |
| Switched to community or online source | 14.8 | 11.0 | 16.7 | 9.0 |
| Change in source of supply among community or online source clients, % | n = 53 | n = 42 | n = 12 | n = 60 |
| Did not change source | 52.8 | 52.4 | 41.7 | 48.3 |
| Switched to public facility | 39.6 | 28.6 | 50.0 | 41.7 |
| Switched to private facility | 7.5 | 19.0 | 8.3 | 10.0 |

This analysis does not include women using LAM at either time point, or implant and IUD users who did not get their method during COVID. Community or online source includes community health worker, outreach event, pharmacy, chemical, or drug shop and ordering on a website, app, or phone.
